# Monocyte-derived APCs are central to the response of PD1 checkpoint blockade and provide a therapeutic target for combination therapy

**DOI:** 10.1136/jitc-2020-000588

**Published:** 2020-07-19

**Authors:** Sjoerd T T Schetters, Ernesto Rodriguez, Laura J W Kruijssen, Matheus H W Crommentuijn, Louis Boon, Jan Van den Bossche, Joke M M Den Haan, Yvette Van Kooyk

**Affiliations:** 1 Molecular Cell Biology and Immunology, Amsterdam Institute for Infection and Immunity, Cancer Center Amsterdam, Amsterdam UMC - Location VUMC, Amsterdam, The Netherlands; 2 Polpharma Biologics, Utrecht, The Netherlands

**Keywords:** costimulatory and inhibitory T-cell receptors, dendritic cells, immunotherapy, programmed cell death 1 receptor, tumor microenvironment

## Abstract

**Background:**

PD1 immune checkpoint blockade (αPD1 ICB) has shown unparalleled success in treating many types of cancer. However, response to treatment does not always lead to tumor rejection. While αPD1 ICB relies on cytotoxic CD8^+^ T cells, antigen-presenting cells (APCs) at the tumor site are also needed for costimulation of tumor-infiltrating lymphocytes (TILs). It is still unclear how these APCs develop and function before and during αPD1 ICB or how they are associated with tumor rejection.

**Methods:**

Here, we used B16 mouse melanoma and MC38 colorectal carcinoma tumor models, which show differential responses to αPD1 ICB. The immune composition of ICB insensitive B16 and sensitive MC38 were extensively investigated using multi-parameter flow cytometry and unsupervised clustering and trajectory analyses. We additionally analyzed existing single cell RNA sequencing data of the myeloid compartment of patients with melanoma undergoing αPD1 ICB. Lastly, we investigated the effect of CD40 agonistic antibody on the tumor-infiltrating monocyte-derived cells during αPD1 ICB.

**Results:**

We show that monocyte-derived dendritic cells (moDCs) express high levels of costimulatory molecules and are correlated with effector TILs in the tumor microenvironment (TME) after αPD1 ICB only in responding mouse tumor models. Tumor-resident moDCs showed distinct differentiation from monocytes in both mouse and human tumors. We further confirmed significant enrichment of tumor-resident differentiated moDCs in patients with melanoma responding to αPD1 ICB therapy compared with non-responding patients. Moreover, moDCs could be targeted by agonistic anti-CD40 antibody, supporting moDC differentiation, effector T-cell expansion and anti-tumor immunity.

**Conclusion:**

The combined analysis of myeloid and lymphoid populations in the TME during successful and non-successful PD1 ICB led to the discovery of monocyte-to-DC differentiation linked to expanding T-cell populations. This differentiation was found in patients during ICB, which was significantly higher during successful ICB. The finding of tumor-infiltrating monocytes and differentiating moDCs as druggable target for rational combination therapy opens new avenues of anti-tumor therapy design.

## Introduction

The inhibitory checkpoint receptor programmed cell death 1 (PD1) was found to dampen tumor-reactive cytotoxic CD8^+^ T cells in the tumor microenvironment (TME).^1^ Many forms of cancer cell types express the ligand PDL1, which can be upregulated in the presence of local immune activation.^2^ Antibodies blocking this PD1/PDL1 interaction, a class of therapy called immune checkpoint blockade (ICB), show impressive patient survival benefit in a wide range of cancers.^3–6^ Tumor intrinsic properties such as mutational load,^4 7^ neoantigen load,^8 9^ metabolism^10^ and genetic subtype affect the response to ICB. However, still a minority of patients show a clinical response and a large portion of patients exhibit tumor cell extrinsic acquired or adaptive resistance, even after initially effective ICB.^11^ Therefore, understanding the mechanism of action of PD1 checkpoint blockade is crucial to facilitate rational design of combination therapies. The effector phase of the anti-tumor immune response induced by anti-PD1 (αPD1) ICB is dependent on cytotoxic CD8^+^ T cells recognizing tumor cells expressing peptide–MHC class I complexes.^12^ Surprisingly, the main target on PD1 receptor engagement has been shown not to be the T-cell receptor but instead CD28 co-stimulatory receptor.^13 14^ PD1 recruits SHP2, which in turn dephosphorylates CD28, preventing tumor-infiltrating lymphocyte (TIL) activation. Activation of CD28 on CD8^+^ TILs is needed to support TIL proliferation and anti-tumor immunity. Therefore, the mode of action for PD-1 blocking antibodies has been proposed to be by reducing the brake of PD1 on the costimulatory capacity of CD28. The co-stimulatory receptor CD28 can be triggered by the members of the B7 family of immunoglobulin superfamily, including CD80 and CD86.^15 16^ Since CD80 and CD86 are primarily expressed by antigen-presenting cells (APCs), successful αPD1 ICB is now thought to rely on the presence of CD80/CD86-expressing APCs, capable of re-stimulating tumor-reactive CD8^+^ T cells.^17^ A wide variety of APCs have been identified in human and murine tumors.^18^ However, it is still unclear how tumor-resident APCs arise during αPD1 ICB and how they are connected to the effector cell populations (ie, NK, T cells) capable of tumor cell killing. Moreover, little is known on the immunological predisposition in the tumor and whether this already defines the αPD1 ICB responsiveness, as the local TME is already instigated by different immune responding cells.

To investigate the lymphoid and myeloid cell accumulation in αPD1 ICB responsive and non-responsive settings, we used the mouse models MC38 and B16 and included the pretreatment stage of both tumor models. This allowed us to analyze and compare changes in the immune compartment in both tumors before or after αPD1 treatment and revealed which tumor-resident APC is crucial to affect the increase in tumor-specific effector cells during αPD1 treatment. The differentiation of intra-tumoral monocytes into APCs was further analyzed using single-cell RNA sequencing data and trajectory analyses. As such, monocytes differentiating in the tumor are a critical component of the PD1 ICB anti-tumor immune response and provide a target for rational combination therapy, including agonistic anti-CD40 antibodies, and can be designed to increase its success rate in survival benefit.

## Methods

### Mice

Wild-type C57BL/6 mice were bred at the animal facility of VU University (Amsterdam, Netherlands) under specific pathogen-free conditions and included in experiments at 8–16 weeks of age. Female and male mice were equally divided among groups, unless stated otherwise. All experiments were approved by the Animal Experiments Committee of the VU University and performed in accordance with national and international guidelines and regulations.

### Tumor models and treatment

Tumor cells (mouse MC38 colorectal carcinoma and B16 melanoma) were cultured in DMEM up to 90% confluency in T75/T175 flasks before injection. Mice were anesthetized using 2% isoflurane, flanks were shaved and tumor cells in serum-free medium were subcutaneously injected in a volume of 50 µL. Tumor size was measured using digital calipers every 2 days in a double-blind manner. Total tumor volume was calculated using the formula 4/3×π×abc (a=width of the tumor/2, b=length/2 and c=the average of a and b). Mice were sacrificed when tumor exceeded 800 mm^3^ or at the end of the study (day 23). Treatment using antagonistic PD1 antibody (clone RMP1–14, in house, endotoxin free) or isotype control antibody (250 µg per mouse per injection in PBS) was started at day 9 after tumor cell injection. Agonistic anti-CD40 antibody (clone 1C10, 100 µg per mouse per injection in PBS) was administered together with antagonistic PD1 similarly, for a maximum of two injections. After death, tumors and spleens were carefully isolated for further processing.

### Tissue digestion, sample preparation and antibody staining

Tumors and spleens were cut small using sterile scissors in 385 µg/mL liberase TL (2WU) and incubated at 37°C for 30 min. Enzymes were deactivated using ice-cold RPMI 1640 complete medium (10% FCS, 1% 50 U/mL penicillin, 50 µg/mL streptomycin, HEPES/EDTA) and incubated at 4°C for 10 min while shaking. After digestion, cells were run through a 100 µm cell strainer and extensively washed before FACS staining. Spleens were additionally resuspended in ACK lysis buffer (Thermo Fisher), incubated for 3 min at room temperature (RT) and resuspended in RPMI complete medium. All antibody staining was performed using freshly made phosphate buffered saline (PBS)/bovine serum albumin (BSA) 1% at 4°C until 2% paraformaldehyde (PFA) fixation. In short, a maximum of 3×10^6^ cells were collected in a well of a 96-well V-bottom plate for staining. Surface markers were stained for 30 min using primary-labeled antibodies in the presence of 1 µg/mL anti-CD16/CD32 Fc block (Biolegend). For intracellular iNOS staining, cells were washed twice with PBS and fixed using 4% PFA/PBS for 15 min at 4°C. After washing with PBS, cells were permeabilized using 0.5% saponin in PBS/BSA 1% for 15 min and subsequently stained using anti-iNOS antibody in 0.5% saponin/PBS/BSA 1% for 30 min at RT. For FoxP3 nuclear staining, cells were fixed and permeabilized according to the manufacturer's instructions (Foxp3 Transcription Factor Staining kit; Thermo Fisher) and incubated with anti‐Foxp3‐PE antibodies (clone 150D; Biolegend) for 20 min at RT. Samples were acquired within 48 hours of antibody staining. Flow cytometry staining included single-stained and fluorescence-minus-one (FMO) controls for every organ and for every FACS acquisition run to control for day-to-day acquisition variation. For antibodies used in this study, see online supplementary table 3.

10.1136/jitc-2020-000588.supp4 Supplementary data

### Flow cytometry acquisition, data pre-processing and analysis

All flow cytometry experiments were performed at the O2 Flow Facility at Amsterdam UMC (Netherlands) using an X20 Fortessa flow cytometer (BD Biosciences). The cytometer was daily calibrated using CS&T calibration beads (BD Bioscience), and all samples in the study were measured with the same lot number of CS&T calibration beads. For acquisition, all samples were filtered using a 70 µm cell strainer, resuspended in 250 µL and acquisition was performed by a plate loader set at 1.0 µL/s acquisition speed. Flow cytometry data were analyzed first using FlowJo analysis software. First, files were compensated using UltraComp eBeads (Thermo Fisher) microspheres labeled with the appropriate fluorochrome-labeled antibodies. Compensation was additionally verified using FMO controls for every single fluorochrome for every tissue type (equally pooled per group) on experimental samples. First, gating was performed on a stable flow (time vs cell count), subsequently on viability dye-negative/lin-negative/CD45-positive cells and finally on single cells (FSC-A/FSC-H). The resulting cells of all individual samples were concatenated per tissue type and exported per experimental group into an Flow Cytometry Standard (FCS) file and uploaded to the Cytobank online analysis platform (https://www.cytobank.org/) for tSNE and CITRUS analysis.

### Unsupervised clustering analyses of flow cytometry data (tSNE, CITRUS)

Using the ViSNE module of Cytobank, we generated tSNE plots per tissue type based on the following input and analysis settings: (1) for TILs, equal number of single alive CD45^+^CD19^−^CD11b^−^NK1.1^−^CD3^+^ cells (concatenated) per condition used up to 30,000 single cells total, number of iterations=3000, perplexity=50, Theta=0.5; (2) for myeloid cells, all single alive CD45^+^CD19^−^CD3^−^NK1.1^−^ cells (concatenated) per condition used up to 100,000 single myeloid cells total, number of iterations=3000, perplexity=50, Theta=0.5. Next, we identified and manually gated subpopulations as represented by the tSNE clustering analysis, color-coded, and overlaid the subpopulations as represented in the graphs. After defining manual gating strategies, the individual experimental samples were similarly gated in FlowJo and statistics were exported to GraphPad Prism V.7 for visualization and statistics. Marker intensity stainings (geometric mean fluorescence intensity) were normalized using subset-specific and tissue-specific FMO for every single acquisition day. The background gMFI derived from subset-specific and tissue-specific FMO was subtracted from the sample subset-specific and tissue-specific gMFI and plotted in heatmaps using GraphPad Prism V.7.

### Diffusion mapping using destiny

To analyze single-cell trajectories of monocyte differentiation in MC38 tumors, we performed diffusion map as implemented in the R package *destiny*.^19^ Briefly, the SPADE module of Cytobank was used for the definition 20 clusters based in tSNE variables. Compensated. FCS files were exported and loaded in R (V.3.5.1) and arcsinh transformed using the package *flowCore*.^20^ Clusters CD11b^+^Ly6G^−/lo^ were selected for implementation of *DiffusionMap* function using width sigma defined by the function *find_sigmas*, k nearest neighbor=100 and distance=“euclidean”.

### Single-cell RNA sequencing data analysis (tSNE, destiny, Monocle)

Transcripts per million (TPM) normalized data from previously published single-cell RNA-seq of tumor samples from patients with melanoma treated with ICB^21^ (GSE120575) were downloaded from the NCBI Gene Expression Omnibus webpage (https://www.ncbi.nlm.nih.gov/geo/query/acc.cgi?acc=GSE120575). tSNE, as implemented in the R package *Rtsne*,^22^ was performed using the 1000 most variable genes, defined by IQR, perplexity=50, and 5000 iterations. Clusters found in tSNE were classified in different cell populations based in genes described in the original publication. *GSVA* package was used for single-sample gene set enrichment scores based on the cell specific gene signatures defined previously.^23^ Hierarchical clustering was used for the definition of different cell populations within the myeloid cells using the 1000 most variable genes, defined by IQR. Trajectories of monocyte differentiation were analyzed using a diffusion map, in a similar way as explained previously, or by using the *Monocle* package.^24 25^ The package limma was used for the differential gene expression analysis, using each cluster of the myeloid compartment (monocytes, macrophages and moDCs) but also including pDCs.

### Bulk RNA sequencing correlations (gene set, single gene)

RNA-seq data from bulk tumor samples were downloaded applying the function *getGEO* as implemented in the package *GEOquery*, using the GSE ID GSE91061.^26^ Gene set enrichment was performed in a single-sample basis using the *GSVA* package, using custom gene sets or the ones defined previously.^27^ Spearman correlation between each GSVA score or individual gene expression was applied as in the package *psych*.

### Statistics

Statistics were performed using GraphPad Prism V.7 software. For the comparison of two groups, a two-tailed Fisher’s t test was applied. For more than two groups, a one-way analysis-of-variance (ANOVA) was used followed by a Tukey post hoc analysis to compare means between two groups. When two variables define multiple groups, a two-way ANOVA was used followed by a Sidak multiple comparison test. *p<0.05, **p<0.01, ***p<0.001, ***p<0.0001, data represented as mean±SEM.

## Results

### Αnti-PD1 checkpoint blockade changes global intra-tumor immunity independent of therapeutic efficacy in established syngeneic tumors

**Figure 1:**
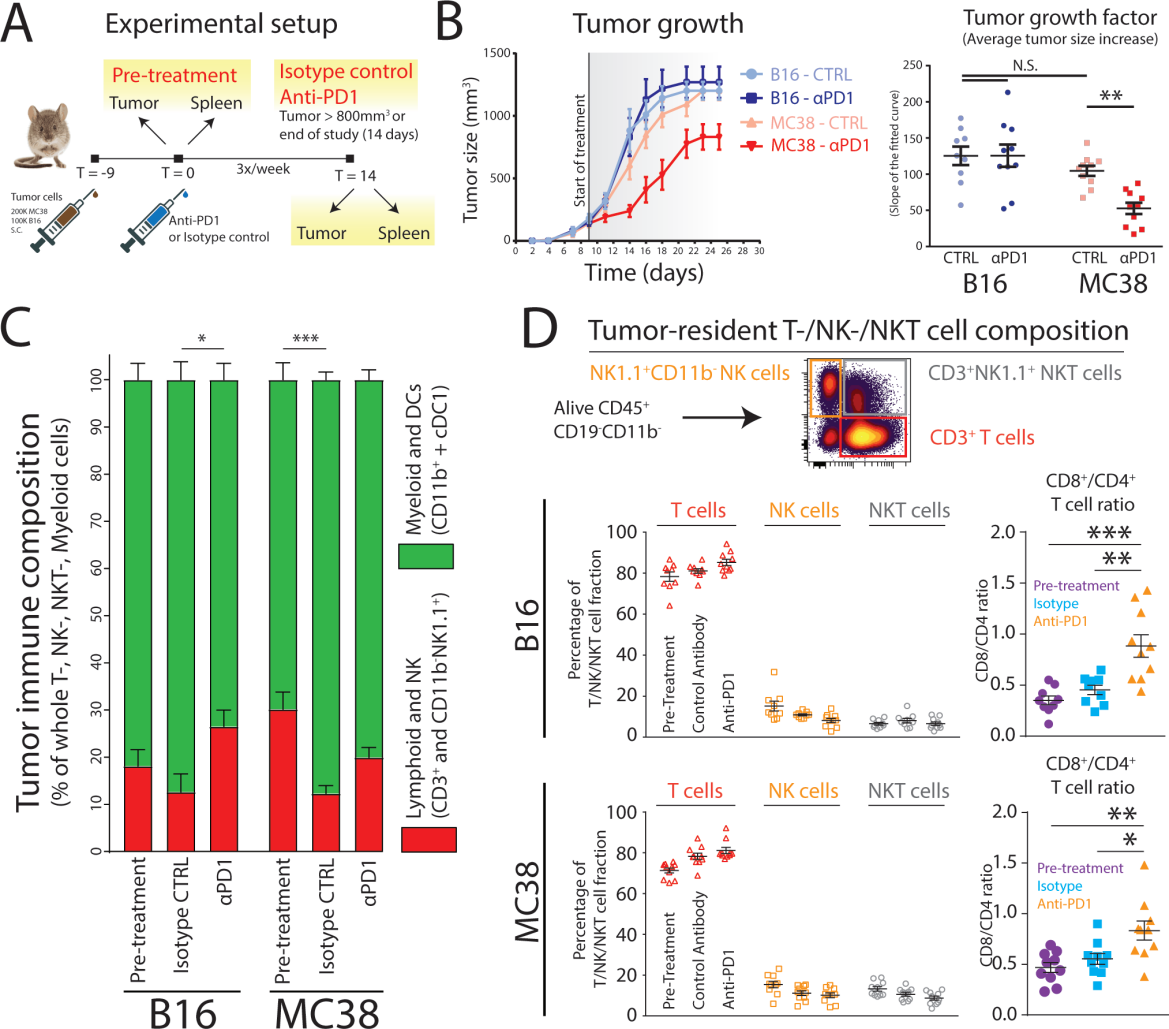
Established MC38 colorectal carcinoma tumors, but not established B16 melanoma tumors, are responsive to αPD1 checkpoint blockade. (A) Syngeneic B16 melanoma or MC38 colon carcinoma tumors were subcutaneously grown for 9 days to similar size, after which treatment was started. (B) Growth curves of B16 and MC38 tumors over time show the difference between B16 and MC38 responsiveness to αPD1 checkpoint blockade. Growth curves were quantified by fitting a linear curve (y=αx+β) and plotting the α (ie, the average growth per day). (C) The immune composition of B16 and MC38 tumors before and after treatment (αPD1 or isotype control) as defined by CD11b^+^ myeloid cells, conventional DC1s (cDC1s), CD3^+^ T cell, CD11b^−^NK1.1^+^ NK/NKT cell compartment. (D) Relative changes in tumor-infiltrating T, NK and NKT cells in B16 and MC38 tumors, as well as differences in the ratio of CD8^+^/CD4^+^ TILs. Data shown as mean±SEM, n=9–10 per group. Statistics performed: two-way (B) or one-way (C, D) ANOVA with Tukey post hoc; *p<0.05, **p<0.01, ***p<0.001, ****p<0.0001. Representative of 2 individual experiments.

To investigate the effect of PD1 checkpoint blockade on the global immune composition of two established syngeneic tumors (αPD1 responsive (MC38) and αPD1 non-responsive (B16)), we devised an experimental setup by tailoring injected cell numbers to equalize tumor size at 9 days after tumor cell injection and before starting αPD1 treatment or isotype control (intraperitoneally 3×250 µg/week; figure 1A, online supplementary figure 1A). Importantly, we included pretreatment groups to identify immunological pre-dispositions prior to ICB. Interestingly, already 2 to 4 days after the first dose of αPD1, there was an evident decrease in tumor growth in established MC38 tumors but not in B16 tumors. To quantify the tumor growth rate over time, we fitted a linear curve to measured tumor sizes, yielding a growth factor per tumor (ie, average tumor size increase per day; online supplementary figure 1B). There was no significant difference in tumor growth between B16 and MC38 tumors and tumor take was 100% (figure 1B). As expected, the tumor growth factor significantly decreased in MC38 tumors treated with αPD1, compared with isotype control antibody, while no changes were observed for B16 tumors (figure 1B). Given that the immune system mediates the response to PD1 ICB, we next questioned whether this was reflected by the tumor-immune composition. Using flow cytometry, we first measured the lymphoid/NK versus myeloid cell/dendritic cell (DC) balance, before and after PD1 treatment and observed an increase in lymphoid/NK cells in both tumor models after αPD1 ICB compared with isotype control (figure 1C). Of note, in any condition tested, the myeloid/DC compartment dominated the TME (figure 1C). Furthermore, subdivision of the CD11b^−^ lymphoid/NK compartment based on expression of CD3 and NK1.1 showed similar changes in T, NK and NKT cells in B16 and MC38 tumors. More specifically, the relative contribution of NK (NK1.1^+^CD3^−^) and NKT (NK1.1^+^CD3^+^) cells decreased over time (αPD1 or isotype control), while the contribution of CD3^+^ TILs increased during tumor growth and after αPD1 treatment (figure 1D). CD11b^+^ NK cells are a subset excluded in this analysis. Importantly, the ratio of CD8^+^ TILs over CD4^+^ TILs significantly increased after αPD1 treatment in both tumor models (figure 1D). Hence, while similar growing B16 and MC38 tumors show increases in CD8:CD4 T-cell ratio, only the MC38 tumor model shows reduced tumor growth, indicating that quantification of overarching immune subsets does not differentiate between responding and non-responding tumor models.

10.1136/jitc-2020-000588.supp2 Supplementary data

### CD11b^+^Ly6C^+^MHCII^+^CD11c^dim^ APCs are the major tumor-resident APC, show differentiation from monocytes and express distinct co-stimulatory and co-inhibitory molecules

**Figure 2:**
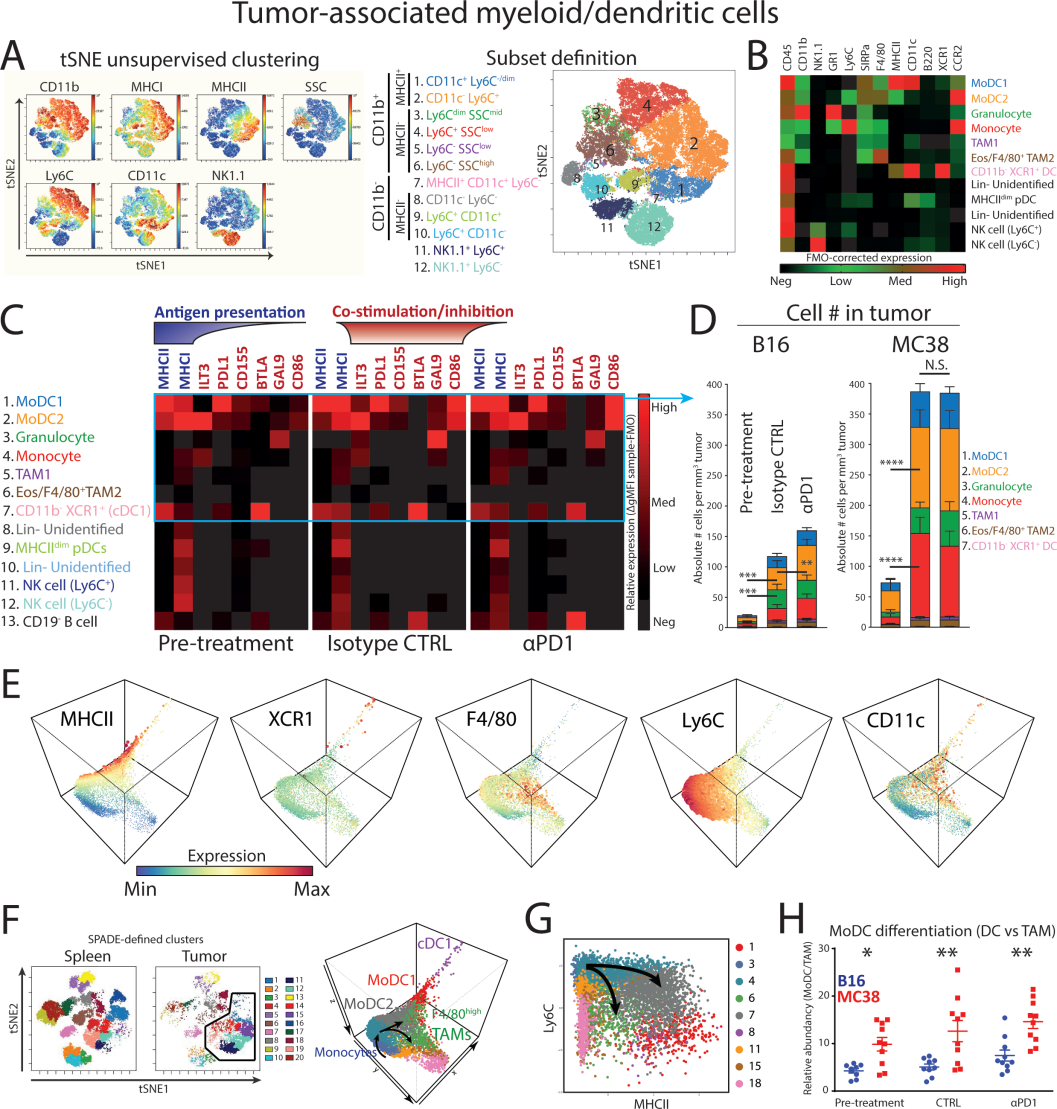
Tumor-associated myeloid cells/dendritic cells in αPD1-responsive tumors and the differentiation from monocytes. (A) tSNE unsupervised clustering of CD45^+^CD3^−^CD19^−^ MC38 tumor-resident cells reveals six CD11b^+^ myeloid cell types (pop 1–6) and one CD11b^−^ APC type (pop 7). (B) Additional high-dimensional flow cytometry experiments verified the identity of tumor-associated dendritic cells (pop 1, 2, 7), granulocytes (G-MDSCs; pop 3), macrophages (pop 5–6), monocytes (pop 4), pDCs (Pop 9), Lin^−^ cells (pop 8,10) and NK1.1^+^ NK cells (pop 11, 12). (C) Marker expression as FMO-corrected gMFI of checkpoint ligands, MHCI and MHCII. (D) Absolute cell number per tumor volume (# cells/tumor volume mm^3^) of myeloid cells and cDCs. (E) 3-dimensional diffusion mapping of myeloid cells (Lin^−^CD11b^+^) and cDC1s (Lin^−^CD11c^+^MHCII^+^XCR1^+^). (F) Unsupervised clustering of tumor-infiltrating and spleen-derived CD45^+^CD19^−^CD3^−^ cells by tSNE and subsequent SPADE clustering on tSNE variables allows unbiased delineation of different cell populations. Diffusion mapping of pre-defined myeloid (CD11b^+^; clusters 1, 3, 4, 6, 7, 11, 15, 18) and cDC1 (MCHII^+^XCR1^+^; cluster 8) clusters in the tumor microenvironment shows differentiation trajectories of tumor-infiltrating monocytes toward moDCs (expressing CD11c, MHC class II and losing Ly6C) or F4/80^high^ TAMs. (G) Diffusion mapping shows similar monocyte differentiation trajectories into moDCs or macrophages based on the Ly6C/MHCII plot, in line with previous reports.^29^ (H) moDC/TAM differentiation balance reveals enriched moDC differentiation in MC38 tumors. Data presented as mean±SEM (n=10 per group). Statistics performed: (D/H) two-way ANOVA with Tukey post hoc; *p<0.05, **p<0.01, ***p<0.001, ****p<0.0001. Graphs are representative of 2 individual experiments.

Considering that the majority of tumor-resident immune cells in both tumor models are myeloid/DCs and potentially provide signals for TIL reactivation and function, we further investigated the heterogeneity of this compartment by multiplex flow cytometry and tSNE-aided gating strategies in both MC38 and B16 tumor models before treatment. Unsupervised clustering by tSNE on CD45^+^CD19^−^CD3^−^ cells based on NK1.1, CD11b, MHCI, MHCII, CD11c, Ly6C combined with classical gating strategies including granularity (side scatter; SSC) clustered 12 populations (figure 2A, online supplementary figure 2A). These subpopulations were found in both tumor models and remained present before and after treatment. The gating strategy and cellular identity was further validated using additional lineage markers, including XCR1, B220, SIRPα, CD11b, Ly6C, CD11c, MHCII, GR1, CCR2, NK1.1, and F4/80 (figure 2B). As a result, we identified six CD11b^+^ myeloid cell populations: tumor-associated macrophages TAM1, monocytes, GR1^high^ neutrophils/PMN-MDSCs, F4/80^+^ TAM2, monocyte-derived dendritic cell populations (moDC1 and 2), in addition to CD11b^−^ DCs (cDC1) (figure 2B). In addition, pDCs, Lin^−^ cells and NK1.1^+^ NK cells could be identified in the tumor-derived CD45^+^CD19^−^CD3^−^CD11b^−^ cells. The GR1^high^ population overlapped with neutrophils present in the spleen (online supplementary figure 2f) and were distinctly clustered from monocytes and monocyte-derived cell populations, suggesting these cells to be neutrophils and not PMN-MDSCs.

We next measured the expression of MHC class I and II complexes and co-stimulatory and co-inhibitory molecules that myeloid cells and APCs employ to interact with T cells. Our results showed that most of the checkpoint ligands were expressed by MHCII^+^CD11c^+^ APCs (figure 2C; pop 1, 2, 7). More specifically, PDL1 (ligand for PD1) was mostly expressed by moDC1 (pop 1) and in intermediate levels by moDC2 (pop 2), as well as CD11b^−^ dendritic cells (pop 7). CD155 (ligand for TIGIT), also known as PVR, was mostly expressed by moDC2s. BTLA (ligand for HVEM) was primarily expressed by cDC1s (pop 7), as reported for tolerogenic peripheral cDC1s.^28^ There was little difference between B16 and MC38 myeloid cells, although moDCs expressed slightly higher levels of CD86 in MC38 pre-treatment conditions (online supplementary figure 2B).

We subsequently quantified the absolute number of cells per tumor volume to properly estimate the absolute effect of myeloid subsets on the TME, what we defined as “immunological pressure”. As a result, monocytes (pop 4; red), moDCs (pop 1; blue and pop 2; orange) and granulocytes (pop 3; green) were shown to comprise the majority of myeloid cells in both the B16 and MC38 tumors (figure 2D). The immunological pressure by myeloid cells increased over time, regardless of tumor type. More specifically, moDC2 and monocytes (ie, orange pop 2 and red pop 4, respectively) significantly increased per tumor volume, but only increased slightly by αPD1 blockade in B16 tumors (figure 2D). The moDC2 was significantly more abundant in MC38 tumors compared with B16 tumors in pre-treatment (online supplementary figure 2C). The previously described cDC1 population (pop 7, pink) contributed approximately 0.3% of the myeloid/DC compartment and did not significantly change in MC38 tumors treated with αPD1 or isotype control (online supplementary figure 2D). These data point toward moDCs as the most abundant APC present in B16 and MC38 syngeneic mouse tumors.

Because monocytes and moDCs are phenotypically closely related in tSNE high-dimensional space and are the major component of the myeloid compartment, we investigated the relationship and putative differentiation by diffusion mapping as applied in the R package *destiny*^19^ (figure 2E). Briefly, myeloid/DC data were used as input in the diffusion map resulting in a three-dimensional diffusion map including the myeloid clusters and the cDC1 cluster. By annotating 20 SPADE-defined clusters derived from tSNE variables, the relation between myeloid and DC subpopulations became evident (figure 2F). Starting with infiltrating monocytes, a bimodal differentiation into MHCII^+^ monocyte-derived DCs (moDCs) or alternatively F4/80^+^ tumor-associated macrophages (F4/80^high^ TAMs) emerged (figure 2F). The cDC1s were at the end point spectrum of the DC differentiation path using these markers. A similar differentiation of monocytes into moDCs or TAMs could be visualized using the Ly6C/MHCII bimodal plot (figure 2G), as previously described for the TS/A mammary adenocarcinoma model.^29^ By quantifying the direction of differentiation (ie, moDCs vs TAMs), monocytes infiltrating MC38 CRC tumors showed a significant preference for differentiation toward moDCs compared with monocytes infiltrating B16 melanoma tumors (figure 2H). Since moDC2 expressed higher levels of CD86 and less PDL1, it suggested to be a more stimulatory (and more abundant) APC compared with the moDC1. In all conditions examined, both moDC2 and moDC1 are present. The trajectory analyses in all dimensions suggest a monocyte-MoDC2-MoDC1 trajectory. However, only the abundance of moDC2 is correlated with the expansion of PD1^−^CD8^+^ T cells, suggesting specific functional properties of the moDC2 that is lost in the moDC1. This transition seems to be associated with the loss of Ly6C, F4/80 and ILT3 and increase of MHCII, CD11c and PDL1, although the functional implications of this exact transition remain to be determined.

In summary, we defined two highly abundant monocyte-derived APC populations differentiated from Ly6C^hi^ monocytes that expressed high levels of co-inhibitory/stimulatory molecules and MHC class I and II molecules. Of interest is the expression of CD86 by moDCs, which could provide co-stimulation to T cells via the CD28 costimulatory receptor known to mediate PD1 blockade efficacy.^13 14^

### PD1-responsive MC38 tumors are enriched in checkpoint-expressing TILs and show increased abundancy correlation with myeloid cells compared with B16 tumors

**Figure 3:**
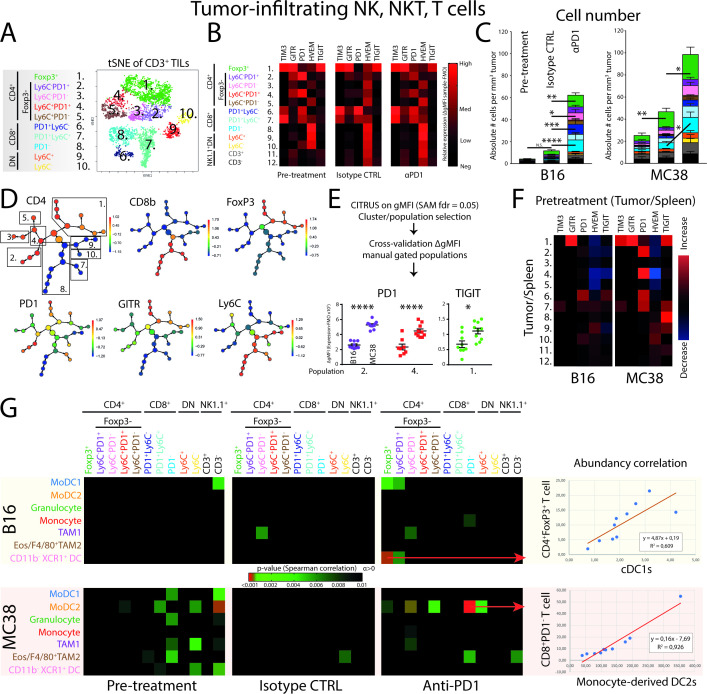
Unsupervised clustering analyses of tumor-infiltrating lymphocytes reveal early establishment of T-cell heterogeneity and specific checkpoint receptor expression. (A) tSNE-guided gating strategy of high-dimensional flow cytometry data derived from all conditions revealed 10 CD45^+^CD11b^−^NK1.1^−^CD3^+^ T-cell populations; five CD4^+^ (pop 1–5), three CD8^+^ (pop 6–8) and two DN (pop 9,10) T-cell populations. Manual gating overlay to the tSNE plot identified TIL clusters (online supplementary figure 3a.) Representative of both B16 and MC38 conditions (online supplementary figure 3b). (B) GMFI corrected with subset-specific FMOs showed TIL subset-specific expression of checkpoint receptors. Data shown of MC38 TILs and representative for both B16 and MC38 models. (C) Absolute cell number per tumor volume (# cells/tumor volume mm^3^) of TILs in both the B16 and MC38 tumors. (D) CITRUS clustering analysis for hierarchical clustering and statistical analysis of differences in expression of checkpoint receptors between B16 and MC38 TILs. (E) Applying significance-analysis-of-microarrays (SAM, fdr=0.05) was applied on the data set yielding significantly different clusters, which were subsequently cross-validated with the FMO-corrected gMFI of manual gated populations. (F) The tumor-driven upregulation of checkpoint receptors was calculated as the paired differences between TIL and peripheral lymphocyte subset equivalent derived from the spleen. (G) Correlation of absolute abundances (number of cells per tumor volume; corrected p value <0.01) of defined myeloid subsets with defined lymphoid subsets. Data presented as mean±SEM (n=10 per group). Statistics performed: (C) two-way ANOVA with Tukey post hoc; (E) unpaired 2-sided Fisher t-test; (G) Spearman correlation analysis. *p<0.05, **p<0.01, ***p<0.001, ****p<0.0001. Graphs are representative of 2 individual experiments.

Next, to see whether changes in myeloid cell populations in the tumor were reflected on effector cell populations, including TILs, NK and NKT cells, we applied high-dimensional flow cytometry to identify major CD3^+^ TIL subsets. Using tSNE unsupervised clustering analysis (on alive CD45^+^CD11b^−^CD3^+^ cells) by expression of CD4, CD8, GITR, FoxP3, PD1, Ly6C identified 10 TIL populations; five CD4^+^ (pop 1–5), three CD8^+^ (pop 6–8) and two double negative (DN) (pop 9, 10) TIL populations (figure 3A). Complemented manual gating strategies corroborated the separation of the tSNE clusters (online supplementary figure 3A). Interestingly, pre-treatment B16 and MC38 solid tumors showed the presence of phenotypically similar T-cell subsets (figure 3B; online supplementary figure 3B). PD1 expression was clearly present on CD4^+^FoxP3^+^ regulatory T cells (Tregs), CD4^+^PD1^+^ conventional TILs and PD1-expressing CD8^+^ T cells, whereas the expression of the exhaustion marker TIM3 was expressed by Tregs and CD8^+^PD1^+^ TILs. The co-inhibitory receptor TIGIT was exclusively expressed by Tregs. Hence, both B16 and MC38 tumor harbored phenotypically suppressive Tregs, exhausted CD8^+^PD1^+^TIM3^+^ TILs and PD1-expressing conventional CD4^+^ TILs. Quantification of absolute numbers of TILs per tumor volume showed a significant increase of CD8^+^PD1^−^ effector TILs and surprisingly Tregs, after PD1 checkpoint blockade (figure 3C). There was also a slight increase in conventional CD4^+^PD1^−^ T cells, although this did not reach statistical significance in treated MC38 tumors. Comparing B16 and MC38 tumors, the absolute abundance was overall higher in MC38 tumors (online supplementary figure 3C).

Since both CD4^+^ and CD8^+^ TILs in pre-treatment conditions expressed PD1, we next aimed to investigate the expression level of PD1 on all subsets of TILs in pretreatment conditions using CITRUS. This analysis allowed the hierarchical clustering and subsequent statistical analysis of stratified subpopulations of high-dimensional flow cytometry data. Using CITRUS, we could recapitulate the 10 identified CD3^+^ TIL populations and perform statistical analysis using significance-analysis-of-microarrays (SAM) (figure 3D). CITRUS identified nodes in which cells express a significant different level of TIM3, GITR, PD1, HVEM or TIGIT between B16 and MC38 TILs. These nodes were cross-validated by manual gating strategies using individual tumor samples and FMO-corrected gMFI was used to validate the differences in expression (figure 3E). As a combined result, two populations of CD4^+^FoxP3^−^PD1^+^ TILs showed increased expression of PD1 in MC38 tumors compared with B16 tumors, while Tregs showed increased expression of TIGIT in MC38 tumors (figure 3E). To validate whether the increased expression of these markers was due to the location of the TILs in the tumor, we compared the expression of markers in splenic counterparts with expression in TILs. Interestingly, the upregulation of PD1 seen in CD4^+^FoxP3^−^PD1^+^ TILs was due to their presence in the tumor and not because of overall systemic changes (figure 3F). These data suggest a significantly higher level of T cell suppression induced by MC38 tumors compared with B16 tumors, most specifically in CD4^+^ TILs.

To investigate whether the accumulation of NK, NKT and T cell effector cell populations are driven by the presence of certain myeloid populations, we correlated the relative abundance (ie, immunological pressure) of NK^−^, NKT^−^ and T cells with myeloid cells/DCs (figure 3G). First, the pre-treatment conditions in MC38 tumors showed more correlations between myeloid and lymphoid/NK cell populations based on abundancy, compared with pre-treatment B16 tumors. Second, this connectivity was lost in end-point tumors treated with isotype control antibodies, suggesting that as the tumor developed lymphoid and myeloid subset accumulated independently of each other. Third, PD1 ICB treatment induced the correlative enrichment of moDC2 with CD4^+^FoxP3^−^PD1^−^ (pop 4), conventional CD4^+^ TILs and CD8^+^PD1^−^ effector TILs (pop 8) only in MC38 tumors (figure 3G). Importantly, since the abundance of moDC2 in MC38 treatment conditions did not increase in PD1 ICB compared with the isotype control, the increase of effector TILs (figure 3G) induced by PD1 checkpoint blockade seemed to be dependent on the presence of moDC2. Although CD8^+^PD1^−^ TILs were also increased in B16 on αPD1 treatment, no connectivity with moDC2 is observed. In B16, the highest abundancy connectivity observed was with cDC1s and FoxP3^+^CD4^+^ regulatory T cells (figure 3G). In summary, both the lymphoid/NK/NKT and the myeloid compartment expands over time, while PD1 ICB mostly leads to expansion of TILs. The expanding TIL populations on PD1 ICB highly correlates with the presence of the moDC2 population only in the MC38 responding tumor model, not in the B16 tumor model or isotype treated MC38 tumors.

### Single cell RNA sequencing of human melanoma biopsies reveals monocyte-derived DCs related to PD1 responsiveness and TIL activity

**Figure 4:**
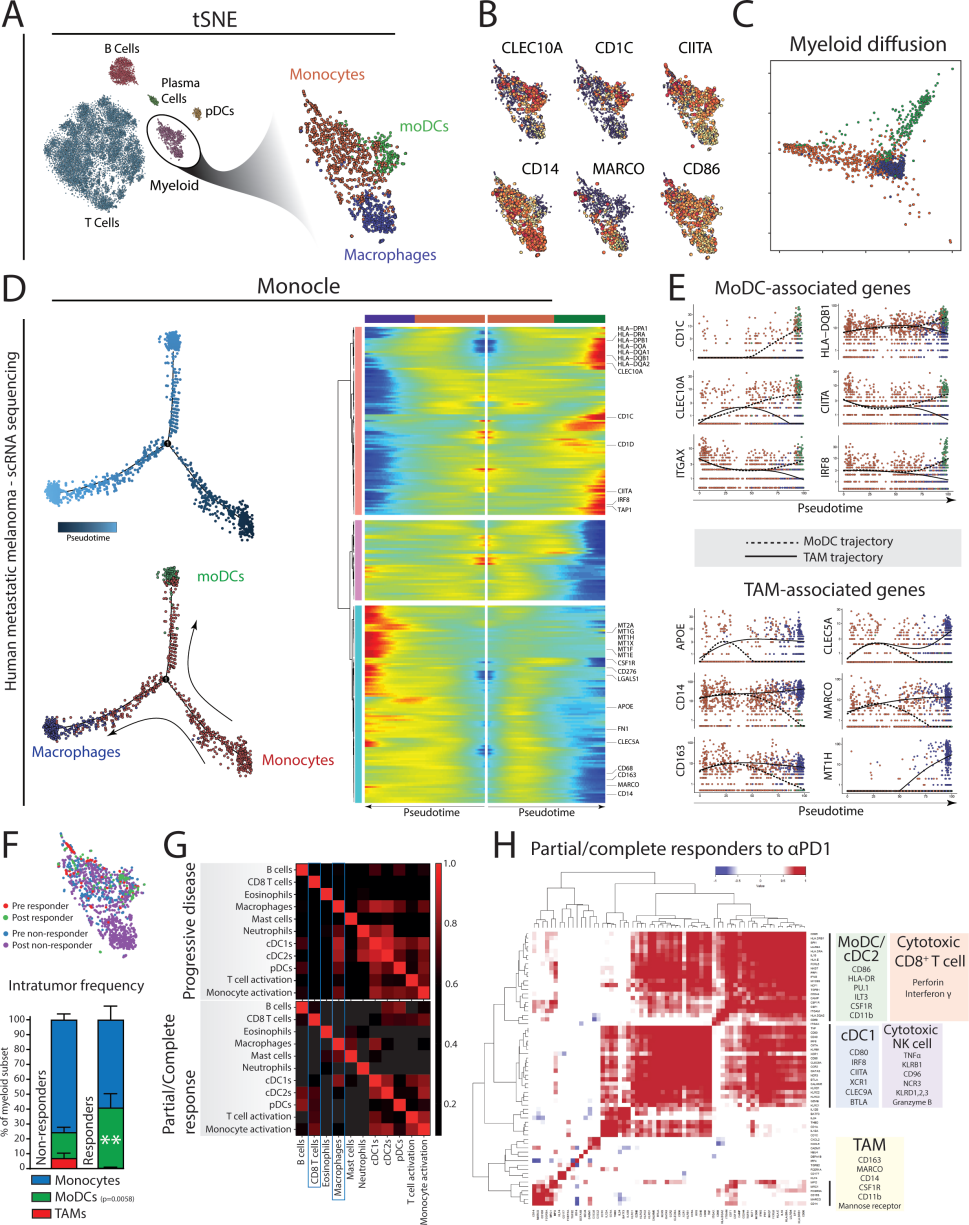
Monocyte-derived cells in human patients with melanoma show a bimodal differentiation pattern related to the therapeutic response of αPD1 therapy. (A) Single-cell RNA sequencing data^21^ of tumor biopsies of patients with metastatic melanoma treated with αPD1 therapy identify myeloid cells, including monocytes, moDCs and macrophages. (B) Expression of several key genes are differentially distributed in the tumor-resident myeloid cells. (C) Bimodal differentiation of monocytes to macrophages or moDCs can be seen using an unsupervised diffusion map. (D) Using the three identified subsets as landmarks, Monocle was used to order cells in pseudotime (the total transcriptional change a cell undergoes as it differentiates along this variable^25^) and allows the visualization of the differentiation process of monocytes to macrophages or dendritic cells. (E) Ordering expression of moDC-related and TAM-related genes of single cells of both moDC and TAM differentiation trajectories in pseudotime. (F) Quantification of monocytes, TAMs and moDCs from tumor biopsies of patients with melanoma either responding or not responding to PD1 checkpoint blockade. (G) Using annotated immune gene sets on bulk transcriptomics of tumor biopsies from patients with advanced melanoma treated with PD1 checkpoint blockade (Riaz *et al* 2017) reveals different gene set correlations (Spearman R) in patients showing a durable response, compared with patients showing progressive disease. (H) Single gene correlation matrix of partial/complete responders indicates co-regulated gene enrichment of moDC/cDC2 genes with genes enriched in cytotoxic CD8^+^ T cell. cDC1-related gene expression was correlated to gene expression related to cytotoxic NK cell activity. Gene expression related to TAMs did not cluster with NK or CD8 T-cell genes, although some genes like CD14 were coregulated with cDC2/moDC genes. Data in (F) presented as mean±SEM (n=30 non-responders; 14 responders). Statistics performed: (F) two-way ANOVA with Sidak post hoc, significance shown of multiple comparison.

Having identified moDC2s as associated with the expansion of effector TILs after successful PD1 ICB, we sought to investigate the infiltrating myeloid cells/DCs in human tumors after PD1 checkpoint blockade. First, we explored single cell RNA sequencing data derived from tumor biopsies of patients with metastatic melanoma.^21^ We were able to identify the myeloid cells similar to the cluster of monocytes/macrophages as identified in the original publication (figure 4A; online supplementary figure 4A).^21^ Subsequently, using hierarchical clustering, we could define monocytes, macrophage and moDCs by expression of *CSF1R*, *CLEC10A*, *MARCO*, *APOE*, *CD14*, *CD163*, *CD1C*, *MAFB* and *CIITA* genes (figure 4B; online supplementary figure 4B). In addition, comparing single cell transcriptomes with previously identified blood DCs further corroborated the identification of DC phenotypes (online supplementary figure 4C). A comparison of the intercellular differential gene expression profiles (see online supplementary table 1 for full gene lists) by Reactome analysis^30^ showed that the transcriptional profile of moDCs is highly enriched in biological pathways related to therapeutic efficacy of checkpoint blockade, including MHC class II antigen presentation, PD-1 signaling, interferon signaling, cytokine signaling and costimulation by the CD28 family (see online supplementary file 1 for complete Reactome analysis reports). Importantly, the most differentially expressed gene in moDCs, cystatin F (CST7), was shown to be highly upregulated in the transition from monocytes to moDCs,^31^ as well as in moDCs derived from peritoneal ascites of patients with cancer.^32^ In addition, CST7 was significantly upregulated in tumor samples from patients with melanoma after treatment with PD1 ICB, specifically in patients responding to the therapy.^26^ Hence, we could identify heterogeneity within the myeloid compartment of tumor biopsies from patients with metastatic melanoma, which include monocytes, macrophages and DCs.

10.1136/jitc-2020-000588.supp3 Supplementary data

10.1136/jitc-2020-000588.supp5 Supplementary data

Unsupervised ordering of the scRNA sequenced myeloid cells by diffusion mapping revealed a monocyte-to-moDC or monocyte-TAM differentiation trajectory (figure 4C). To investigate how key gene expression identifiers change along the monocyte-to-moDC trajectory versus the monocyte-to-macrophage trajectory, we applied Monocle. Monocle allows visualization of progress along several differentiation trajectories as a variable termed pseudotime; the total transcriptional change a cell undergoes as it differentiates along this variable.^25^ We visualized the changes in gene expression of genes associated to macrophages or TAMs (blue) or moDCs (green) along the two trajectories from monocytes (red) in pseudotime (figure 4D). We identified gradual increases of CD163 and APOE early on and a late increase of MARCO, CLEC5A along the macrophage trajectory. Interestingly, the monocyte-to-macrophage pathway shows increased expression of CD276 (B7-H3), an alternative immune checkpoint.^33^ In parallel, we found a gradual increase of MHC class II–related genes (CIITA, HLA-DQB1) and moDC-related genes (CD1C, CLEC10A, CD11c) along the moDC trajectory (figure 4E). These data suggest the presence of a bimodal differentiation trajectory of tumor-infiltrating monocytes into different end points (moDCs or macrophages) in tumor samples from patients with metastatic melanoma. Whether monocytes differentiate with intermediate modes of functioning, as described previously, could not be analyzed with this limited data set and remains to be determined.

Next, we assessed the effect of PD1 checkpoint blockade on this differentiation by quantifying the relative contribution of monocytes, moDCs and TAMs in patients responding to PD1 checkpoint blockade. Importantly, moDCs were significantly more abundant in patients responding to αPD1 therapy compared with patients who did not respond to the therapy (figure 4F). In addition, moDCs from responding patients showed significantly higher levels of IL1β and CCR2 compared with moDCs from non-responsive patients (online supplementary table 2). This suggests that the presence of moDCs is indeed critical for the clinical efficacy of PD1 checkpoint blockade. To further explore the connection between moDCs and the adaptive immune response initiated by PD1 checkpoint blockade further, we analyzed an RNA sequencing data set derived from tumor biopsies of patients with advanced melanoma who were treated with PD1 checkpoint blockade therapy.^26^ Correlating immune-related gene sets within bulk transcriptomic data, we explored the relationship between genes associated with cell subsets and their activation. In tumor tissue from patients who showed a clinical response to PD1 ICB, the expression of the CD8^+^ T-cell gene set correlated with the gene sets of cDC2s, pDCs and monocyte activation (figure 4G). This correlation was absent (or less pronounced) in tumor tissue from patients who showed progressive disease after PD1 ICB. Instead, non-responding patients showed correlation of macrophage gene set with gene sets related to cDC1, cDC2, pDCs and monocyte activation, suggesting that macrophages may regulate other DCs in their response to PD1 ICB and the activation of CD8^+^ T cells (figure 4G). Also, monocytes from non-responding patients expressed significantly higher levels of macrophage-associated genes like CCL2, MARCO and SIGLEC1 compared with monocytes from responding patients, suggesting that non-responsive patients are characterized by a macrophage-prone monocyte infiltrate (online supplementary table 2). Moreover, an unbiased single gene correlation analysis in patients responding to PD1 ICB clusters the expression of moDC/cDC2-related genes (*CSF1R*, *ITGAM*, *LILRB4/ILT3*, *CD86*, *HLA-DB*) with expression of perforin and interferon γ, while cDC1 gene expression (*XCR1*, *BTLA*, *CLEC9A*) clusters with genes related to NK cell cytotoxicity (*GZMB*, *TNF*, *KLRB1*, *CD96*, *NCR3*) (figure 4H). It should be noted that while there was significant overlap between these four clusters, TAM-associated genes did not cluster with these four gene clusters (moDC/cDC2, cDC1, cytotoxic CD8^+^ T cell, cytotoxic NK cell). However, as expected the myeloid lineage genes *CSF1R*, *CSF1* and *ITGAM* were shared between TAM and moDC/cDC2 clusters (figure 4H). In summary, tumors from patients with metastatic melanoma that are successfully treated with PD1 checkpoint blockade show an enrichment of moDCs, which correlate with TIL cytotoxicity.

### Targeting moDCs by agonistic anti-CD40 antibody boosts PD1 checkpoint blockade efficacy, TIL expansion and moDC differentiation into iNOS-producing cells

**Figure 5:**
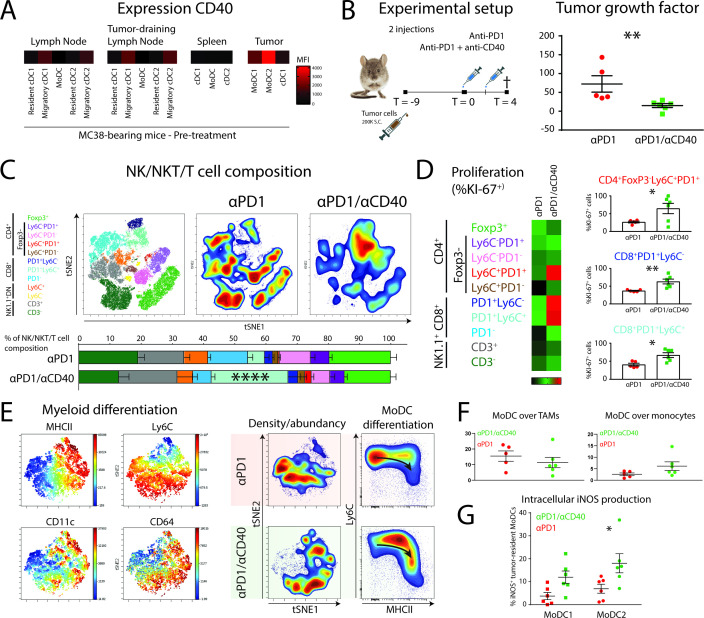
CD40 on tumor-infiltrating MoDCs can be targeted by agonistic anti-CD40 antibodies to augment differentiation of iNOS-producing Tip-DCs and the expansion of effector CD8^+^ and CD4^+^ T cells. (A) Expression levels (FMO-corrected gMFI) of CD40 on DC subsets in MC38-bearing mice at pre-treatment conditions. (B) Mice with established MC38 tumors were either injected with αPD1 or αPD1/anti-CD40 combination therapy at day 9 and day 11 post-tumor cell inoculation. At day 4 after start of the treatment, mice were sacrificed. Tumor growth factor was determined by fitting a linear curve (y=αx+β) to the growth measurements and plotting α (ie, the average growth per day). (C) tSNE unsupervised clustering and manual gating strategies (of CD45^+^CD19^−^CD11b^−^NK1.1^−^) identified the 10 T-cell populations for quantification. (D) T-cell proliferation was measured by intracellular Ki-67 staining in each individual T-cell subset. (E) tSNE unsupervised clustering of myeloid (CD45^+^CD19^−^CD3^−^NK1.1^−^CD11b^+^GR1hi^−^) cells shows differentiation of monocytes driven by agonistic anti-CD40 antibody treatment. (F) Monocyte-to-TAM and monocyte-to-moDC differentiation as indicated by the relative abundance. (G) iNOS production was measured by intracellular staining of iNOS in moDC subsets. Data presented as mean±SEM (n=5 per group). Statistics performed: unpaired 2-sided Fisher t-test (B/D); one-way ANOVA with Tukey post hoc (C); two-way ANOVA with Tukey post hoc (F); *p<0.05, **p<0.01, ***p<0.001, ****p<0.0001.

The observation that moDCs may be central to the successful response to αPD1 ICB prompted us to explore rational combination therapies. Agonistic CD40 antibody has previously been suggested to synergize with αPD1 checkpoint blockade by increasing DC-mediated T-cell priming or by blocking PDL1 upregulation.^34 35^ First, we measured the expression of CD40 on APCs in pre-treatment MC38 tumors, tumor-draining lymph nodes, distal lymph nodes and spleens. MoDCs in the tumor expressed the highest level of CD40 (figure 5A), although expression can also be found on migratory cDCs in lymph nodes. Second, we started treatment of αPD1 therapy or αPD1/anti-CD40 therapy at day 9 (1 injection at day 9 and 1 injection at day 11) and sacrificed the mice 4 days after the start of treatment (figure 5B). We found a significant decrease in tumor growth when anti-CD40 was combined with αPD1 ICB (figure 5B). The tumors were further analyzed by high-dimensional flow cytometry to delineate changes in myeloid and lymphoid subpopulations. The change in lymphoid populations was pronounced and entailed a significant increase of the CD8^+^PD1^+^Ly6C^+^ TILs when αPD1 was combined with anti-CD40 (figure 5C). To investigate whether the increase in CD8^+^PD1^+^Ly6C^+^ TILs was because of local proliferation, we used intracellular staining for the proliferation marker Ki-67. Indeed, CD8^+^PD1^+^Ly6C^+^ TILs showed increased expression of Ki-67 suggestive of local proliferation (figure 5D). Interestingly, also CD8^+^PD1^+^Ly6C^−^ and a small population of CD4^+^Ly6C^+^PD1^+^ TILs showed significant increases in KI67^+^ cells. In line with these findings, the expansion of a CD8^+^PD1^+^ memory-like TILs on immunotherapy has been described recently.^36^ To investigate the effect of CD40 agonists on moDCs during PD1 ICB, we looked into the differentiation of myeloid cell types in treated tumors. TSNE unsupervised clustering and typical Ly6C/MHCII “waterfall plots” showed that the combination of αPD1 with anti-CD40 increased the differentiation from monocytes into moDCs (figure 5E). However, the increase in differentiation towards moDCs was at the expense of monocytes, not TAMs (figure 5F). To investigate whether moDCs located in tumors can be triggered by CD40 ligation to upregulate iNOS and support intratumoral T-cell expansion,^37^ we stained intracellular iNOS. Interestingly, we indeed found a significant increase of iNOS levels in moDCs, with the largest upregulation in moDC2s when αPD1 ICB was combined with anti-CD40 agonistic antobody (figure 5F). Hence, moDCs form are rational target for combination therapy of CD40 agonists with αPD1 ICB by facilitating expression of iNOS by moDCs and supporting T-cell expansion.

## Discussion

PD1 ICB has proven to be a therapeutic intervention with clinical efficacy in a wide variety of cancer types. However, a large portion of patients with cancer does not benefit or shows secondary resistance after an initial response. Indeed, the molecular and cellular components needed for successful therapy are not completely understood, preventing patient selection or rational combination therapy design. Pre-clinical mouse models of αPD1 ICB can be used to gain insight in changing immune populations related to successful anti-tumor immunity. However, many studies start treatment before injected cell properly established large tumors.^38 39^ Using two syngeneic mouse tumor models with similar large sizes and growth rates, but differential susceptibility to αPD1 ICB, we first show that heterogeneity in the T-cell response is established before treatment and αPD1 treatment significantly increases the number of effector CD8^+^PD1^−^ TILs. The expression of PD1 on expanding or infiltrating TILs on ICB is a matter of debate since both PD1^−^ and PD1^+^ CD8^+^ TILs have been shown to mediate the anti-tumor effect of PD1 ICB.^36 40^ It is possible that this discrepancy may be due to differences in expansion of peripherally recruited PD1^−^ CD8^+^ T cells and pre-existing PD1^+^ CD8^+^ TILs. While we have investigated pre-treatment conditions, mouse and human time points in this study are based on long-term treatment effects. Initial CD8^+^ TIL expansion may be mediated by a PD1-expressing subset, whereas long-term CD8^+^ T-cell expansion may be supported by peripherally recruited PD1^−^CD8^+^ T cells.

Extensive work using mass cytometry analysis of T-cell responses by Wei and colleagues has previously shown that the T-cell response to αPD1 therapy in these models is relatively comparable. However, before treatment, PD1 ICB-responsive MC38 tumors show higher levels of PD1 on CD4^+^ TILs and higher levels of the inhibitory receptor TIGIT on CD4^+^FoxP3^+^ regulatory TILs, suggestive of a more extensive suppressed TIL microenvironment. This is accompanied by a significant correlative abundancy between myeloid and lymphoid cell populations in MC38 tumors only. Indeed, the absolute numbers of lymphoid and myeloid cells per tumor volume is significantly higher in MC38 tumors before treatment and are indicative of a typical “hot” tumor microenvironment with susceptibility to PD1 ICB.^41^ Interestingly, after tumors are treated with αPD1 checkpoint, both tumor models exhibit increases in effector TIL populations, but only the MC38 tumor model shows decreased tumor growth. Since inhibitory signaling of PD1 acts directly on the CD28 costimulatory receptor on T cells,^13 14^ we investigated the expression of CD86 (CD28 ligand) on tumor-resident myeloid cells and APCs. Expression of CD86 was the highest on CD11b^+^Ly6C^+^CD11c^+^MHCII^+^ moDC2 that form the major myeloid component in the TME suggesting these cells to be prime candidates to mediate CD28-dependent PD1 blockade. Recently, it has been shown that APCs are critical for the local re-stimulation of effector CD8^+^ T cells after αPD1 therapy,^42^ although the subtype of APC responsible remained to be clearly defined. CD11b^+^Ly6C^+^CD11c^+^MHCII^+^ moDCs comprise the majority of tumor-resident APCs in both B16 and MC38 tumors, and they are correlated in abundancy with effector TILs only in successfully αPD1-treated conditions only in MC38. It should be noted that the same moDCs were present in non-responsive B16 tumors, however, at a lower abundance per tumor volume compared with MC38 tumors. Hence, the interplay/connectivity between monocyte-derived APCs expressing co-stimulatory molecules and lymphoid populations is a typical feature of successful checkpoint blockade.

We further investigated the source of monocyte-derived dendritic cells by multiplex flow cytometry and unsupervised diffusion modeling. Monocytes infiltrating the αPD1 treated tumor seem to differentiate via a bimodal differentiation pathway, either differentiation into MHC class II-expressing moDCs or F4/80^high^ tumor-associated macrophages (TAMs). A similar bimodal differentiation pathway of tumor-infiltrating monocytes has been shown in the TS/A mammary adenocarcinoma model^29^ and is proposed to underlie the efficacy of ICB in a recent study by Gubin and colleagues.^43^ Moreover, moDCs have been shown to reactivate adoptively transferred tumor-specific CD8^+^ T cells and induce anti-tumor immunity.^37^ Similarly, chemotherapy mediates recruitment of monocytes that differentiate to moDCs to activate T cells as APCs.^44^ To verify monocyte-differentiation patterns in human tumors, we analyzed single-cell RNA sequencing data from patients with advanced melanoma treated with αPD1.^21^ We could distinguish *CSF1R*-expressing myeloid cells and differentiation toward TAMs with increasing expression of *MARCO*, *CD163* and *APOE*, or moDCs with increasing expression of *CLEC10A*, *HLA-DR*, *CST7* and *CD1C*. The differentiation of monocytes into moDCs has previously been shown to depend on the aryl hydrocarbon receptor (AHR) and the differentiation into macrophages on the transcription factor MAFB.^45^ Indeed, melanoma-resident moDCs express higher levels of AHR, while TAMs express higher levels of the MAFB transcription factor. MoDCs express higher levels of CD86 and the CIITA transcription factor, driving MHC class II protein expression. Tumor-resident monocyte-derived cells with increased activation status and antigen presentation machinery have also been detected by single cell RNA sequencing in human breast cancer tumors.^46^ Alternatively, increased differentiation into TAMs, instead of moDCs, may result in resistance to ICB. For example, we find the TAM differentiation pathway involves increased expression of CD276 (B7-H3), a known immune checkpoint molecule related to impaired CD8^+^ T-cell anti-tumor cytotoxicity.^33^ Indeed, CD276 blockade enhanced the effect of PDL1 or PD1 ICB in mouse tumor models.^33 47^

Monocyte differentiation to moDCs seems to be prevalent in both mouse and human tumors. We show that moDCs are significantly increased in patients responding to αPD1 ICB compared with the patients who do not respond to therapy. Furthermore, moDCs in αPD1 treated patients show enrichment for genes related to effective anti-tumor immunity, including MHC class II antigen presentation, interferon gamma signaling and CD28 co-stimulation. It has been shown before that moDCs derived from ascites of patients with ovarian cancer were differentiated from monocytes.^32^ These tumor-derived moDCs were capable of cross-presenting exogenous antigens to CD8^+^ T cells, and they also provided co-stimulatory signals like CD86.^32^ Indeed, we find “monocyte activation” and “cDC2” gene sets correlated with CD8^+^ T cells only in patients with melanoma exhibiting a partial or complete response to αPD1 treatment, compared with patients who show progressive disease. Moreover, hierarchical correlation clustering of myeloid-related and lymphoid-related genes showed a close association of moDC/cDC2 genes with effector cytokines perforin and interferon gamma, while cDC1-related genes associated with cytotoxic NK cell genes. The association of cDC1 with NK cells has been shown before to mediate an alternative anti-tumor immune response.^48 49^ An outstanding question is whether monocytes infiltrating tumors during PD1 ICB in humans exhibit intermediate differentiation states with distinct functions or even locations. While the data set by Sade-Feldman and colleagues provided critical information on overall cellular trajectories of myeloid cells, the data were underpowered to make any conclusions about intermediate states. Interestingly, the transcriptional activator *CIITA*, which drives expression of MHC class II, is significantly higher expressed in monocytes from patients responding to PD1 ICB, compared with non-responding patients (online supplementary table 2). Since the frequency of CD14^+^CD16^−^MHCII^high^ monocytes before PD1 ICB treatment was a strong predictor of progression-free and overall survival in patients with metastatic melanoma,^50^ the study of monocytes and their differentiation capacity in tumors remain of critical importance.

The identification of the APC that provides re-stimulation in the TME on αPD1 treatment is of relevance when rational combination therapy is considered. Indeed, tumor-associated moDCs express druggable CD40 in MC38 tumors, as well as human ovarian cancer ascites.^32^ We show that monocyte-derived APC can be targeted with an agonistic anti-CD40 antibody to support anti-tumor CD8^+^ T-cell responses and subsequent tumor regression. Monocyte-derived APC produce iNOS in this context and dendritic cells in the TME have previously been shown to produce several effector molecules that augment local anti-tumor CD8^+^ T-cell responses, including iNOS^37^ and IL-12.^42^ Alternatively, CSF1R has been shown to reduce or reprogram suppressive TAMs in the TME^51 52^ and support αPD1 and anti-CTLA4 ICB therapy.^53 54^ Increased moDC differentiation from recruited monocytes after CSF1R may explain these observations since CSF1 is a major driver of the macrophage differentiation fate.^55 56^ Indeed, blocking CSF1R signaling favors differentiation of monocytes into MHCII^high^ TAMs.^57^ Also, CSF1 production by CD8^+^ T cells has been shown to induce resistance to αPD1 therapy by increasing the number of immune suppressive TAMs.^58^

A limitation of this study is the correlative nature of the findings. The identified tumor-resident APC subset shows differentiation from monocytes and represents most likely an inflammatory driven-decision of infiltrating monocytes. Therefore, tools that deplete monocytes or knockout genes in a monocyte-specific manner will also impact tumor-associated macrophages. We have targeted the CD40-expressing moDCs with agonistic CD40 antibody to boost PD1 ICB. Although myeloid cells are assumed to be the main target of agonistic CD40 antibodies,^59^ we cannot exclude the possibility that agonistic CD40 boosts priming of CD8^+^ and CD4^+^ T cells in the tumor-draining lymph node.^60 61^ Alternatively, agonistic CD40 may induce tumoricidal tumor-infiltrating monocytes that deplete tumor stroma, resulting in tumor regression in a T cell–independent manner.^62^ In terms of our data, increased inflammation, induced independently of moDCs, may affect the differentiation of monocytes into moDCs. Also, it is still unclear where the DC–T cell interaction blocked by PD1 ICB occurs and which DC subsets are critical. For example, Fransen and colleagues have shown the importance of the tumor-draining lymph node in anti-PD1 therapy in MC38 tumors.^63^ In addition, pharmacological blocking the egress of lymphocytes from the lymph nodes during PD1 ICB negate the therapeutic effect, showing the critical role of T-cell expansion in the lymph node.^63 64^ The presence of moDCs was correlated with the effector PD1^-^CD8^+^ T-cell population, possibly infiltrating after activation in the lymph nodes. Whether this moDC-related CD8^+^ T-cell population is the critical IFNγ-producing cytotoxic subset is likely, but not verified in this study. Virtually all current dendritic cell depletion models, both genetic and cytotoxic, do not make distinctions between the tumor-draining lymph node and the tumor. Therefore, the exact role of priming, co-stimulation and local restimulation by APC subsets will need to be carefully investigated in both space and time.^65^ Whether monocyte infiltration and differentiation is an essential part of this response remains to be determined in more detail and will likely depend on the composition of the TME and the peripheral immune status before treatment. Also, while the immunological diversity of syngeneic mouse models allows the investigation of differential therapeutic responses, anti-tumor immune responses may have different modes of action in different tumor types or models.^66^ It is possible that the mode of action of PD1 blockade may be different in other mouse tumor models and should be investigated with care. Lastly, we have not included the role of B cells in this study, which have been shown to affect the anti-tumor immune response in certain models.^67^ In human patients, tumor-infiltrating B cells were shown to associate in tertiary lymphoid structures,^68^ a feature poorly modeled in acute mouse tumor models.

In conclusion, we show that the presence of moDCs in the TME is central to successful PD1 ICB expressing co-stimulatory molecules for reactivation of effector TILs. Moreover, we show that additional re-stimulation of moDCs by anti-CD40 boosts moDC differentiation and iNOS production, and effector TIL expansion. Our study and the study by Gubin and colleagues support the paradigm that tumor-infiltrating monocytes drive downstream macrophage polarization during ICB, instead of tumor-resident repolarization of existing macrophage subsets. This provides a novel approach of targeting infiltrating monocytes that may increase the success rate of anti-tumor responses in patients who respond poorly to PD1 ICB monotherapy.

10.1136/jitc-2020-000588.supp1 Supplementary data
