# Supplementary material for: Monocyte-derived APCs are central to the response of PD1 checkpoint blockade and provide a therapeutic target for combination therapy
Source: J Immunother Cancer. 2020 Jul 19;8(2):e000588. doi: 10.1136/jitc-2020-000588 (PMC7371367; doi:10.1136/jitc-2020-000588)
Supplement: Supplementary data [file jitc-2020-000588supp002.pdf]

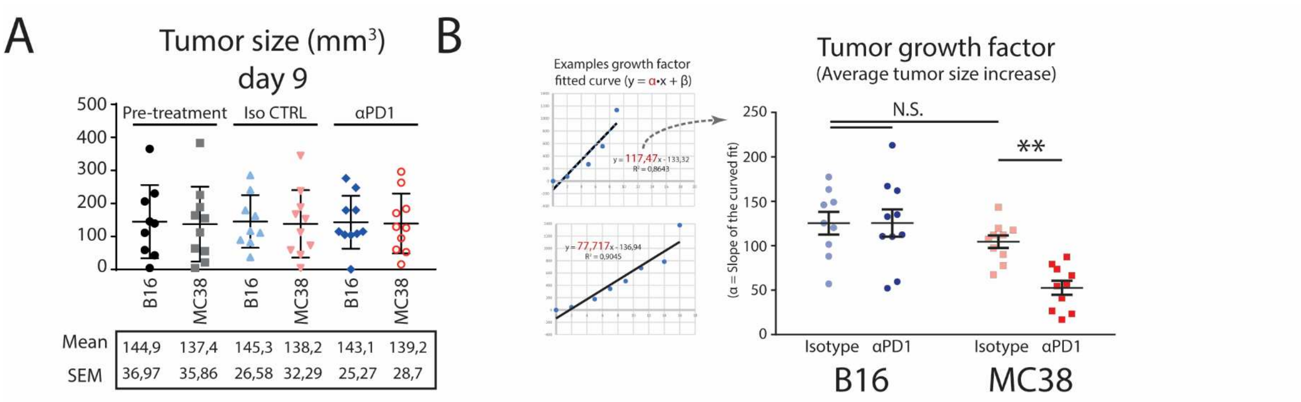


**Supplementary Figure 1 | A.** Tumor growth at 9 days after 1-2 x $10^5$ tumor cells injected subcutaneously in the flank, before tumor-bearing mice were divided into groups. **B.** Tumor growth could be approximated by the steepness (α) of the y = αx + β of tumor size measurements ($2^{nd}$ value > 0).

**Supplementary Figure 2 (Next Page) | A.** Manual gating strategy of alive CD45+CD19-CD3- tumor-resident cells and overlaid to the tSNE unsupervised clustering plot using the same markers. **B.** Expression of MHC class I and -II, and co-stimulatory/co-inhibitory molecules on tumor-resident myeloid cells across all tumor types and conditions. Mean of FMO-corrected gMFI of 9-10 mice per group shown. **C.** Absolute number of myeloid cells in B16 and MC38 in pre-treatment conditions. Mean+-SEM and Fisher t-test. **D.** Composition of MC38 tumors with outlined focus on cDC1s. **E.** The presence of conventional type 1 dendritic cells before and after PD1 ICB in the MC38 tumor model. **F.** tSNE unsupervised clustering of tumor and spleen samples from MC38 tumor-bearing mice. tSNE coordinates were used to define 20 clusters by SPADE analysis. Myeloid/DC clusters 1, 3, 4, 6, 7, 8, 11, 15, 18 were used for trajectory analysis as depicted in figure 2F and 2G. All data from N = 9-10 per group and representative of 2 individual experiments, mean +-SEM.

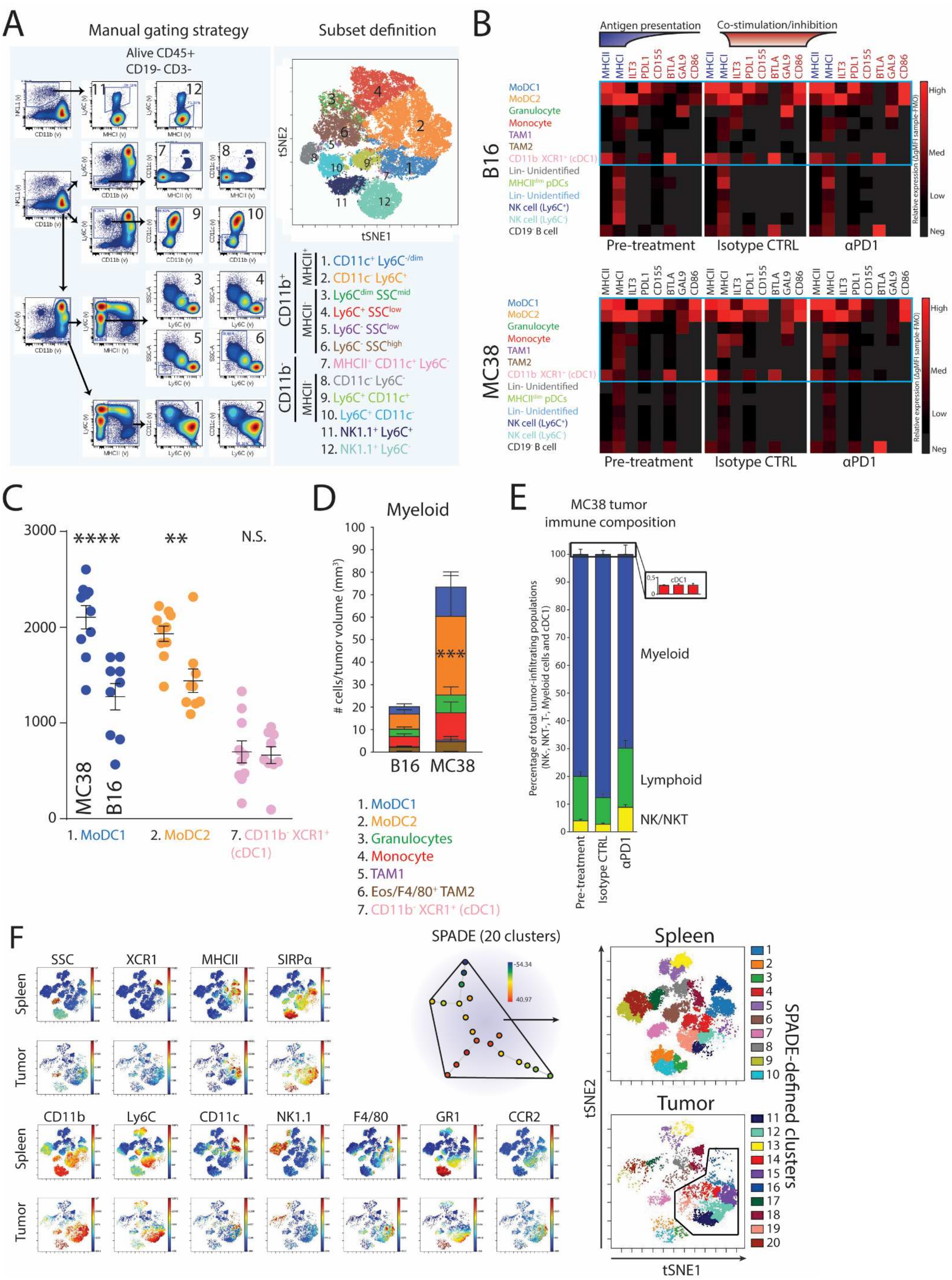

A
Manual gating strategy
Subset definition
Alive CD45+
CD19- CD3-
tSNE2
tSNE1
CD11b+
MHCII+
MHCII-
CD11b-
1. CD11c+ Ly6C-/dim
2. CD11c- Ly6C+
3. Ly6Cdim SSCmid
4. Ly6C+ SSClow
5. Ly6C- SSClow
6. Ly6C- SSChigh
7. MHCII+ CD11c+ Ly6C-
8. CD11c- Ly6C-
9. Ly6C+ CD11c+
10. Ly6C+ CD11c-
11. NK1.1+ Ly6C+
12. NK1.1+ Ly6C-
B
Antigen presentation
Co-stimulation/inhibition
MHCII MHCI ILT3 PDL1 CD155 BTLA GAL9 CD86
B16
MC38
MoDC1
MoDC2
Granulocyte
Monocyte
TAM1
TAM2
CD11b- XCR1+ (cDC1)
Lin- Unidentified
MHCIIdim pDCs
Lin- Unidentified
NK cell (Ly6C+)
NK cell (Ly6C-)
CD19- B cell
Pre-treatment
Isotype CTRL
αPD1
High
Med
Low
Neg
Relative expression (ΔgMFI sample-FMO)
C
**** ** N.S.
MC38
B16
1. MoDC1
2. MoDC2
7. CD11b- XCR1+ (cDC1)
D
Myeloid
# cells/tumor volume (mm3)
B16 MC38
***
1. MoDC1
2. MoDC2
3. Granulocytes
4. Monocyte
5. TAM1
6. Eos/F4/80+ TAM2
7. CD11b- XCR1+ (cDC1)
E
MC38 tumor
immune composition
Percentage of total tumor-infiltrating populations (NK-, NKT-, T-, Myeloid cells and cDC1)
cDC1
Myeloid
Lymphoid
NK/NKT
Pre-treatment
Isotype CTRL
αPD1
F
SSC XCR1 MHCII SIRPα
CD11b Ly6C CD11c NK1.1 F4/80 GR1 CCR2
Spleen
Tumor
SPADE (20 clusters)
SPADE-defined clusters
tSNE1
tSNE2

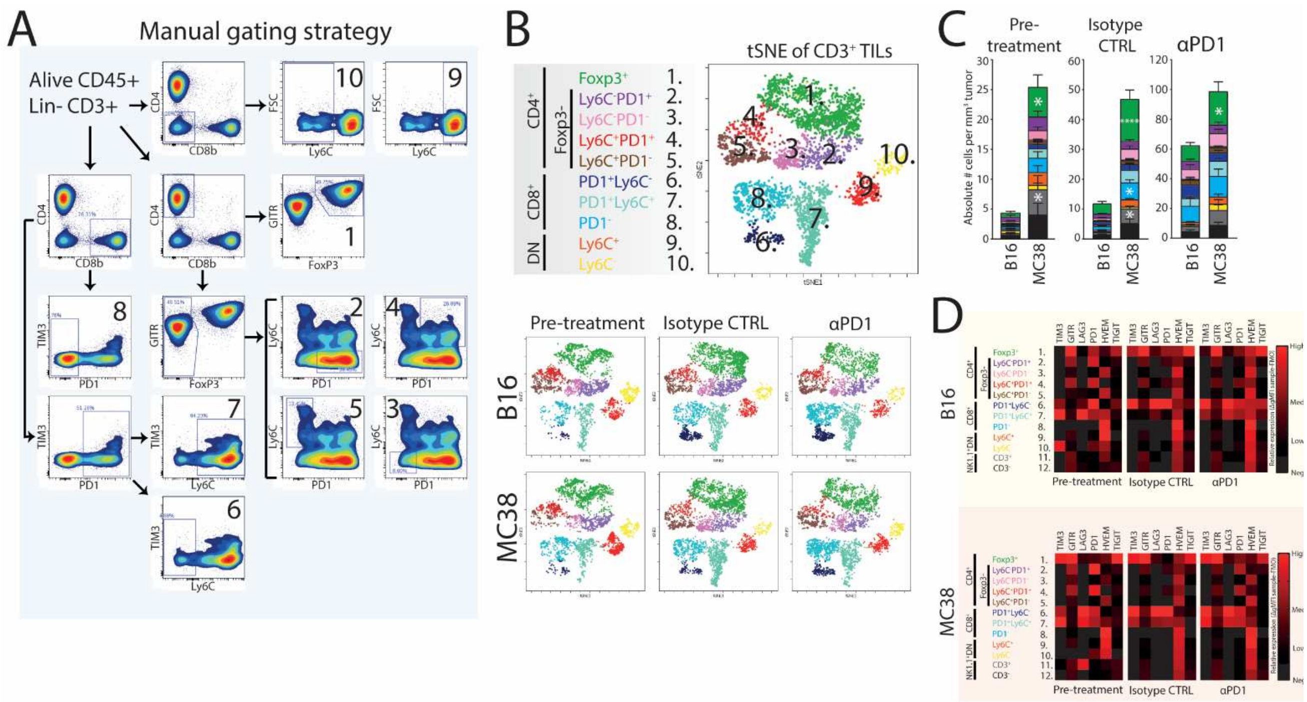


**Supplementary Figure 3 | A.** Manual gating strategy of alive CD45$^{+}$CD19$^{-}$CD11b$^{-}$CD3$^{+}$ tumor-resident cells. **B.** Manual gated populations overlaid to the tSNE unsupervised clustering plot using the same markers, across all tumors and conditions. Data shown are concatenated files from 6 mice per group. **C.** Quantification as absolute number of identified cells per tumor volume of B16 and MC 38 tumors. **D.** Subset-specific expression of co-stimulatory/inhibitory receptors in B16 and MC38 tumors. Mean +-SEM and Fisher t-test. All data from N = 9-10 per group and representative of 2 individual experiments.

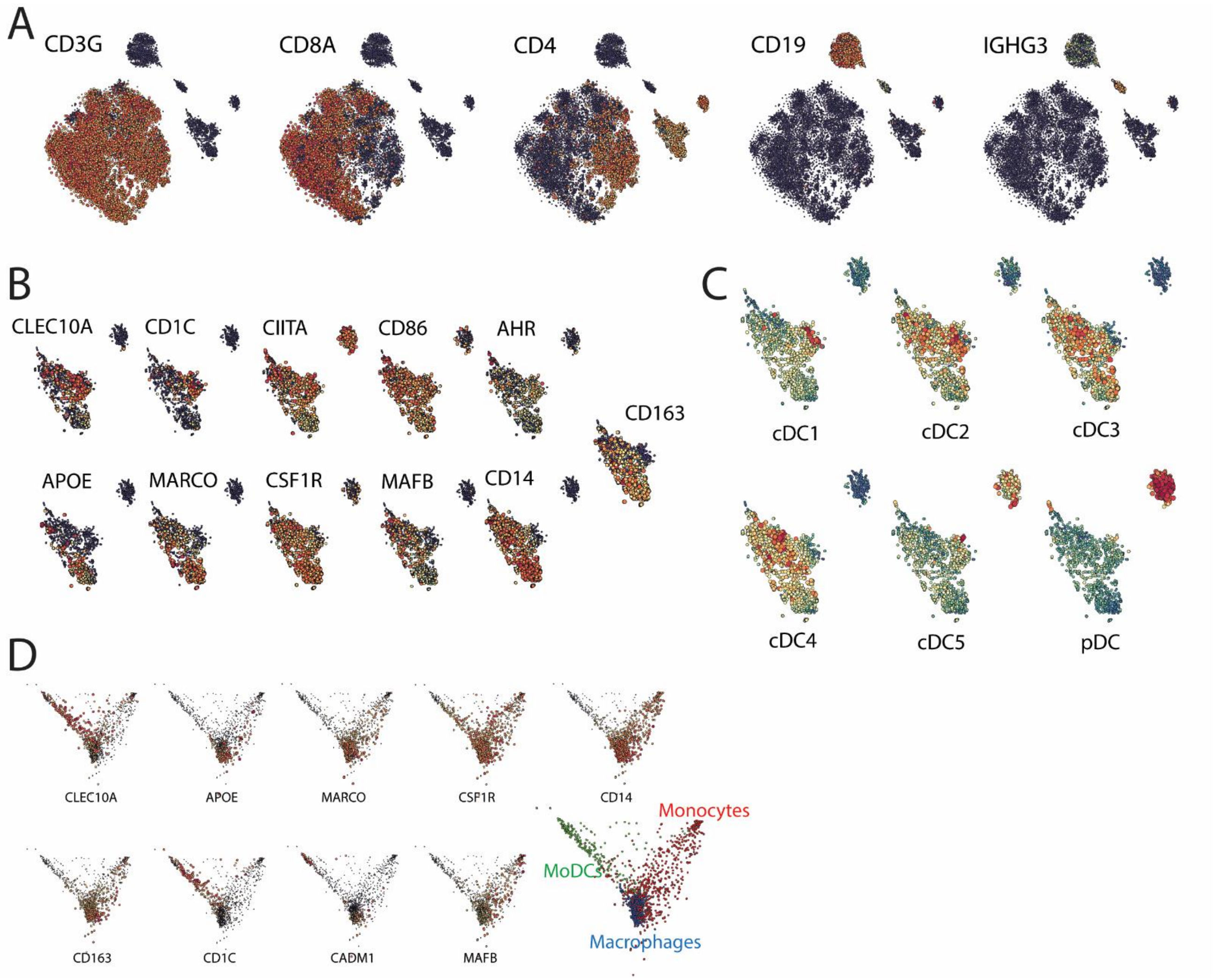


**Supplementary Figure 4 | A.** Expression of canonical markers on tSNE of scRNA sequencing data from melanoma patients tumor biopsies[21]. **B.** Expression of key myeloid genes within myeloid compartment. **C.** Comparison of single-sample gene set enrichment scores based on the cell-specific gene signatures defined by Villani and colleagues[23]. **D.** Expression of key myeloid genes expression within the myeloid compartment and visualized within diffusion plots showing monocytes, macrophages and moDCs.
